# Rapid Detection of Pathogenic Bacteria by the Naked Eye

**DOI:** 10.3390/bios11090317

**Published:** 2021-09-06

**Authors:** Karthikeyan Kandasamy, Miftakhul Jannatin, Yu-Chie Chen

**Affiliations:** 1 Department of Applied Chemistry, National Chiao Tung University, Hsinchu 300, Taiwan; mirokarthik.c@nycu.edu.tw (K.K.); miftakhulj.ac07g@nctu.edu.tw (M.J.); 2 Department of Applied Chemistry, National Yang Ming Chiao Tung University, Hsinchu 300, Taiwan

**Keywords:** endogenous enzymatic reactions, *Escherichia coli*, *Staphylococcus aureus*, cotton swab, tetramethyl benzidine, naked eye detection

## Abstract

*Escherichia coli* O157:H7 and *Staphylococcus aureus* are common pathogens. Gram-negative bacteria, such as *E. coli*, contain high concentrations of endogenous peroxidases, whereas Gram-positive bacteria, such as *S. aureus*, possess abundant endogenous catalases. Colorless 3,5,3′,5′-tetramethyl benzidine (TMB) changes to blue oxidized TMB in the presence of *E. coli* and a low concentration of H_2_O_2_ (e.g., ~11 mM) at pH of 3. Moreover, visible air bubbles containing oxygen are generated after *S. aureus* reacts with H_2_O_2_ at a high concentration (e.g., 180 mM) at pH of 3. A novel method for rapidly detecting the presence of bacteria on the surfaces of samples, on the basis of these two endogenous enzymatic reactions, was explored. Briefly, a cotton swab was used for collecting bacteria from the surfaces of samples, such as tomatoes and door handles, then two-step endogenous enzymatic reactions were carried out. In the first step, a cotton swab containing bacteria was immersed in a reagent comprising H_2_O_2_ (11.2 mM) and TMB for 25 min. In the second step, the swab was dipped further in H_2_O_2_ (180 mM) at pH 3 for 5 min. Results showed that the presence of Gram-negative bacteria, such as *E. coli* with a cell number of ≥ ~10^5^, and Gram-positive bacteria, such as *S. aureus* with a cell number of ≥ ~10^6^, can be visually confirmed according to the appearance of the blue color in the swab and the formation of air bubbles in the reagent solution, respectively, within ~30 min. To improve visual sensitivity, we dipped the swab carrying the bacteria in a vial containing a growth broth, incubated it for ~4 h, and carried out the two-stage reaction steps. Results showed that bluish swabs resulting from the presence of *E. coli* O157: H7 with initial cell numbers of ≥ ~34 were obtained, whereas air bubbles were visible in the samples containing *S. aureus* with initial cell numbers of ≥ ~8.5 × 10^3^.

## 1. Introduction

Foodborne illnesses caused by pathogenic bacteria can result in diarrhea, abdominal pain, nausea, fever, and even death [1,2,3,4,5], and these pathogens cannot be easily identified on the basis of these symptoms [3]. Foodborne illnesses are life-threatening to the elderly, children, or newborn babies because of their weak immune systems [4,5]. Bacterial infections caused by pathogenic bacteria, including *Escherichia coli* [6], *Shigella soney* [7], *Listeria monocytogenes* [8], *Salmonella* spp. [9], and *Staphylococcus aureus* [10], demonstrate high mortality rates [11]. Agricultural products, such as vegetables, are commonly linked to bacterial contamination [12,13,14,15,16]. Hundreds of thousands of foodborne pathogenic infection cases are reported every year [14]. Vegetables contaminated by bacteria, such as *E. coli* O157:H7, a Gram-negative bacterium, have led to several foodborne disease outbreaks [13]. *S. aureus*, a Gram-positive bacterium, is another common pathogen that can cause foodborne illnesses [15]. In addition to causing food poisoning, bacteria such as *E. coli* J96 can cause infectious diseases, such as urinary tract infections [16]. *S. aureus* is also a common pathogen that causes skin infections [17].

In general, different types of antibiotics are used to treat illnesses resulting from Gram-positive and Gram-negative bacteria. Given that current industrial washing treatments for fruits and vegetables cannot guarantee 100% pathogen-free products [18], determining whether bacterial contamination originates from Gram-positive or Gram-negative bacteria is vital. Meaningful information necessary to medical treatment can be obtained by determining whether infections or contaminations are caused by either Gram-positive or Gram-negative bacteria. However, traditional bioassays that require overnight culture take at least 3–5 days to identify bacteria in real-world samples [19,20]. Moreover, although molecular diagnostic tools, such as real-time polymerase chain reactions, have high sensitivity, their execution needs well-trained personnel to reduce the possibility of obtaining false-positive results [21]. Immunoassays are fast and sensitive, but their applicability is limited by the availability of antibodies for diverse bacterial targets [22]. Although Gram-staining can be easily used in identifying Gram-positive and Gram-negative bacteria, their results require examination via optical microscopy. Furthermore, false-positive Gram-staining results may be obtained because of high decolorization, excessive heat during fixation, insufficient crystal violet concentration, and Gram-staining-resistant bacteria [23,24,25]. Moreover, it is usually recommended that bacteria should be freshly harvested from overnight or extended 18–48 h cultures before Gram-staining [26]. Thus, a screening method that is based on naked-eye detection and has speed, reliability, and high sensitivity without requiring overnight culture is desirable.

Most Gram-negative bacteria, such as *E. coli*, contain high amounts of peroxidases [27], which have been used in catalyzing the conversion of colorless 3,5,3′,5′-tetramethyl benzidine (TMB) into visible oxidized TMB with a blue color in the presence of H_2_O_2_ [26,27,28,29]. Supporting Information Scheme S1 in the Supplementary Materials shows the peroxidase reaction when TMB is used as a substrate in the presence of H_2_O_2_. At this point, the color of the resultant reaction is blue, whereas the reaction color changes to yellow when sulfuric acid is added to stop the reaction [30]. Owing to the existence of endogenous peroxidases in *E. coli* O157:H7, peroxidase-based colorimetric reactions have generally been used in determining the presence of *E. coli* O157:H7 under standard buffer conditions at the lowest detectable concentration of ~10^5^ cfu mL^−1^ [31]. However, most existing methods that use peroxidases for the visualization of the presence of Gram-negative bacteria still require overnight culture before peroxidase reaction tests can be carried out [32,33,34,35,36]. Moreover, Gram-positive bacteria, such as *S. aureus*, contain abundant catalases [37] that can catalyze the generation of oxygen in the presence of H_2_O_2_ [37] (SI Scheme S1). Satisfactory results, in which the limit of detection (LOD) of *E. coli* O157: H7 is as low as ~10^3^–10^4^ cfu mL^−1^, can be obtained after 4–6 h of bacterial culture before PCR analysis [38]. However, PCR analysis is time-consuming and labor-intensive because tedious sample pretreatment steps must be completed before the analysis can be performed [38].

Cotton swabs are useful tools for collecting trace samples from the surfaces of target samples [39]. Thus, we developed a rapid sensing method for detecting Gram-negative and Gram-positive, catalase-positive bacteria in samples by using cotton swabs as the tool. In this method, a swab is used as a sampling and sensing probe, and endogenous enzymatic reactions derived from target bacteria are used for distinguishing the presence of bacteria. The feasibility of using the swab-based testing approach in distinguishing Gram-negative bacteria from Gram-positive bacteria was demonstrated. The optimal experimental conditions were then examined, and cherry tomatoes and door handles contaminated by bacteria were used as real samples.

## 2. Methods

### 2.1. Materials and Reagents

Monopotassium phosphate, di-sodium phosphate, TMB, and phosphoric acid were purchased from Sigma-Aldrich (St. Louis, MO, USA). Potassium chloride was purchased from Fluka (Muskegon, MI, USA). Sodium dihydrogen phosphate hydrate was purchased from Mallinckrodt (St. Louis, MO, USA). Hydrochloric acid and sodium hydroxide were obtained from J. T. Baker (Phillipsburg, NJ, USA). Hydrogen peroxide was purchased from Showa (Tokyo, Japan). Cotton swabs were obtained from a local shop. Agarose was purchased from Amresco (Solon, OH, USA). Tryptic soy broth (TSB) and yeast extract (Y) were purchased from Becton Dickinson (Franklin Lakes, NJ, USA), whereas the Luria-Bertani (LB) powder was purchased from Neogen (Lansing, MI, USA). *E. coli* J96 was kindly provided by Dr. James Johnson (Minneapolis Veterans Affairs Medical Center and the University of Minnesota, USA). *Klebsiella pneumoniae*, *Pseudomonas aeruginosa*, *Staphylococcus aureus*, *Streptococcus pyogenes,* and *Enterococcus faecalis* were collected from the patients in Hualien Tzu-Chi Hospital and kindly provided by Prof. P.-J. Tsai (National Cheng-Kung University, Taiwan). *E. coli* O157:H7 (BCRC 13085), *Bacillus cereus* (BCRC 17427), and *Aspergillus niger* (BCRC30130) were purchased from the Bioresource Collection and Research Center (Hsinchu, Taiwan). Cherry tomatoes were purchased from a local market.

### 2.2. Instrumentation

All the ultraviolet-visible (UV-Vis) absorption spectra were obtained using a Cary 50 UV-Vis absorption spectrophotometer from Varian (Melbourne, Australia). Cell images were obtained using an Eclipse 80i fluorescent microscope from Nikon (Tokyo, Japan).

### 2.3. Preparation of Bacterial Samples

All the Gram-positive and Gram-negative bacteria used in this study were Risk Group 2 pathogens. Thus, they were prepared in a Biosafety Level 2 laboratory. Gram-negative bacteria, including *E. coli* O157:H7, *K. pneumoniae*, and *P. aeruginosa*, were used as the model bacteria and cultured in LB broth at 37 °C for 12 h. LB broth (10 mL) was prepared by dissolving LB powder (10 g) in deionized water (400 mL). Gram-positive bacteria, including *S. aureus*, *E. faecalis*, *B. cereus*, and *S. pyogenes,* were selected as model bacteria and cultured in TSBY broth (10 mL) at 37 °C for 12 h. The TSBY broth was prepared by dissolving TSB (12 g) and Y (2 g) in deionized water (400 mL). The resultant bacterial samples (10 mL) were centrifuged at 3750× *g* (rotor radius: 93 mm) for 10 min. The precipitated bacterial cells were rinsed with phosphate-buffered saline (PBS) solution (pH 7, 1 mL × 3) under centrifugation at 3750× *g* for 10 min. PBS was prepared by dissolving sodium chloride (400 mg), potassium chloride (10 mg), disodium hydrogen phosphate (57.5 mg), and potassium dihydrogen phosphate (12 mg) in deionized water (50 mL). The pH of the solution was then adjusted to 7. Stock bacterial suspension was prepared in PBS (pH 7) with optical density (OD) at the wavelength of 600 nm (OD_600_) of ~1. Bacterial samples with different concentrations were prepared with serial dilutions from the stock suspension.

### 2.4. Endogenous Peroxidase and Catalase Reactions of Bacteria

The endogenous peroxidase reactions of bacteria were analyzed by reacting model bacteria at a given concentration (e.g., OD_600_ of ~1) prepared in the phosphate buffer (pH 3, 0.2 mL) with H_2_O_2_ at different concentrations (5.6–2890 mM) and TMB (1.25 mM). Phosphate buffer at pH 3 was prepared by adding monosodium dihydrogen phosphate hydrate (53 mg) and disodium hydrogen phosphate pentahydrate (165 mg) in deionized water (50 mL). The pH of the solution was then adjusted using phosphoric acid. The mixture containing bacteria and reagents with a low concentration of H_2_O_2_ was left standing for 25 min during peroxidase reactions and subsequently for catalase reaction, by adding H_2_O_2_ at a high concentration for another 5 min. The resultant sample was examined by the naked eye and UV–Vis absorption spectroscopy.

### 2.5. A Two-Step Method for Distinguishing Gram-Positive from Gram-Negative Bacteria

Distinguishing bacteria based on their endogenous enzymatic reactions was performed by reacting model bacteria with H_2_O_2_ in the presence of TMB. Model bacteria, including Gram-positive and Gram-negative bacteria with an OD_600_ of ~1, were prepared in the PBS buffer at pH 7. A cotton swab was used to sample the bacterial solution (10 µL) followed by immersing the swab in the reagent (50 µL) containing H_2_O_2_ (11.2 mM) and TMB (1.25 mM), prepared in the phosphate buffer at pH 3. The sample stood for 25 min to allow the reaction to be completed. A bluish color appeared on the resultant swab if the sample contained Gram-negative bacteria, whereas bubbles might be observed if trace Gram-positive bacteria were present in the sample. If no bubbles were observed in the sample, the sample was further supplemented with H_2_O_2_ (180 mM, 0.1 mL) prepared in phosphate buffer at pH 3.0 for 5 min. The resultant samples were examined by the naked eye and a photograph was taken with a camera.

### 2.6. Using Cherry Tomatoes as the Simulated Real Sample

Cherry tomatoes smeared with model bacteria were used as the simulated real samples. *E. coli* O157:H7 samples with different concentrations were prepared from the stock bacterial sample with OD_600_ of 1 (~6.8 × 10^8^ cfu mL^−1^), via a serial dilution with the PBS buffer at pH 7. The as-prepared bacterial samples (50 µL) were spread on the surface of the cherry tomato. After drying, a cotton swab was imbued with the phosphate buffer at pH 3 (10 µL), followed by picking up bacteria from the surface of the as-prepared cherry tomato. The resultant swab was dipped into the droplet (50 µL) containing H_2_O_2_ (11.2 mM) and TMB (1.25 mM) at pH 3. After reacting at room temperature for 25 min, the swab was examined by the naked eye, and a photograph was taken with a camera. These results were used as the standards for comparison with the results obtained from the samples prepared in the following way. That is, another three replicated samples (50 µL) containing *E. coli* O157: H7 (~6.8 × 10^7^ cfu mL^−1^) were individually smeared on the surface of three cherry tomatoes by an inoculation loop. The cherry tomatoes were then dried in an oven at 37 °C for 30 min. After drying, the sample from the cherry tomato was detected following the experimental steps stated above.

Alternatively, to improve detection sensitivity, the resultant cotton swab that was obtained after picking up bacteria from the surface of the sample was dipped in a vial containing nutrient medium, such as LB broth (0.4 mL), for ~4 h. The resultant bacterial cells in the vial were separated through centrifugation at 3750× *g* (rotor radius: 93 mm) for 5 min. The bacterial cells were resuspended in the phosphate buffer (pH 3, 0.2 mL) containing H_2_O_2_ (11.2 mM) and TMB (1.25 mM), followed by incubation at room temperature for 25 min. The resultant samples were treated with sulfuric acid (2 M, 2 µL) to stop the reaction. The resultant samples were then examined using UV–Vis absorption spectroscopy.

### 2.7. Detection of Bacteria from Door Handles

Door handles contaminated with model bacteria, such as *E. coli* J96 and *S. aureus,* were prepared and used to simulate real-world samples. Bacterial samples were prepared by serially diluting the stock bacterial samples at OD_600_ of 1 (*E. coli* J96 (OD_600_ of 1 = ~5.5 × 10^8^ cfu mL^−1^); *S. aureus* samples (OD_600_ of 1 = ~1.7 × 10^9^ cfu mL^−1^)) were diluted to a given concentration. The samples, including *E. coli* J96 (~5.4 × 10^6^ cfu mL^−1^), *S. aureus* (~1.7 × 10^9^ cfu mL^−1^), *S. aureus* (~1.7 × 10^7^ cfu mL^−1^), and a mixture of *S. aureus* (~1.7 × 10^8^ cfu mL^−1^) and *E. coli* J96 (~5.4 × 10^6^ cfu mL^−1^), were prepared. The as-prepared bacterial samples (50 µL) were individually spread on the surfaces of door handles. The samples were then dried at room temperature. After drying, a phosphate buffer (pH 3, 10 µL) was deposited on the surfaces of the door handle and swabbed with a cotton swab. The resultant swab was immersed into a reagent (50 µL) containing H_2_O_2_ (at a concentration of 11.2 mM) and TMB (1.25 mM) at pH 3. After 25 min, the cotton swab was examined by the naked eye and a photograph was taken with a camera. The cotton swab was further dipped into a reagent containing H_2_O_2_ (180 mM, 0.1 mL) at pH 3 for another 5 min. The sample was then examined by the naked eye, and a photograph was taken with a camera.

In addition, we also directly collected samples from three door handles in the restroom of our building. Each time, we used two cotton swabs to collect samples from the same door handle. One swab was used for the two-step screening test using the method shown above. The other cotton swab was inoculated on an LB agar plate for 14-hour incubation at 37 °C.

## 3. Results and Discussion

### 3.1. Endogenous Peroxidase Reactions Derived from Bacterial Samples

**Figure 1:**
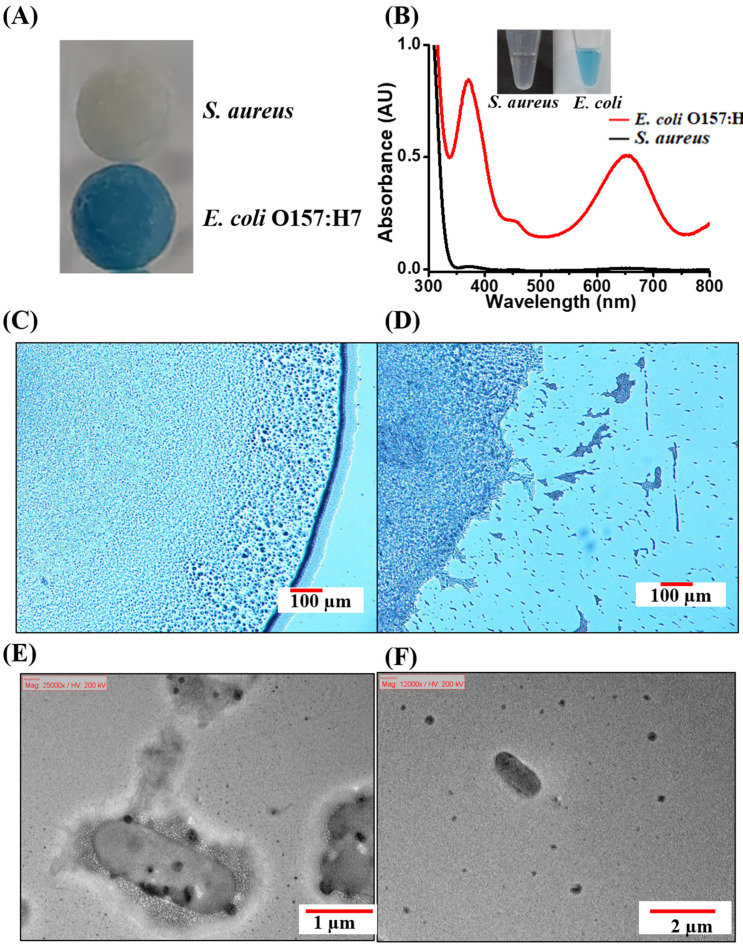
(**A**) Photographs of the cotton swabs obtained after sampling the bacterial samples (50 µL) containing *E. coli* O157: H7 (OD_600_ = ~1) and *S. aureus* (OD_600_ = ~1), after reacting with the reagent (50 µL) containing TMB (1.25 mM) and H_2_O_2_ (11.2 mM) at pH 3 for 25 min. (**B**) UV-Vis absorption spectra of the bacterial samples (0.2 mL) including *E. coli* O157: H7 (OD_600_ = 1) and *S. aureus* (OD_600_ = 1) obtained after reaction with TMB and hydrogen peroxide (11.2 mM) at pH 3 for 25 min, followed by the reaction with additional hydrogen peroxide (7.2 M, 5 µL) for another 5 min. The inset shows the photographs of the resultant samples. Representative microscopic images of *E. coli* O157: H7 (**C**) without and (**D**) with the addition of H_2_O_2_ (11.2 mM, 2 µL) at pH 3. The bacterial samples were stained with methylene blue (1 mM, 2 µL) for 2 min before investigation by the optical microscope. (**E**,**F**) Corresponding TEM images of the samples shown in panels (**C**,**D**).

Given that the goal of this study was to use endogenous enzymatic reactions derived from bacteria for distinguishing Gram-negative bacteria from Gram-positive bacteria, two common pathogenic bacteria, *S. aureus* and *E. coli* O157: H7, were initially selected as the models for investigation. TMB was used as the substrate. Supporting Information Scheme S1 shows the peroxidase and catalase reactions. Figure 1A shows the photograph of the cotton swabs obtained after picking up the model bacteria (50 µL, OD_600_ = 1), following reaction with TMB (1.25 mM) in the presence of H_2_O_2_ (11.2 mM) at pH 3. The cotton swab at the bottom of the photograph was tainted with Gram-negative bacteria (*E. coli* O157:H7), whereas the cotton swab at the top of the photograph was shown after sampling Gram-positive bacteria (*S. aureus*) and remained colorless. Figure 1B shows the UV–Vis absorption spectra obtained after the two model bacteria samples (0.2 mL, OD_600_ = 1) were reacted with TMB (1.25 mM) in the presence of H_2_O_2_ (11.2 mM) prepared in phosphate buffer at pH 3, and the inset shows the corresponding photographs of the samples. The colorimetric response was due to the catalytic activity of the endogenous peroxidase, derived from the bacteria that had reacted with TMB. The blue color was observed only in the presence of Gram-negative bacteria (*E. coli* O157:H7), indicating that the endogenous peroxidase reaction could potentially be applied to distinguish Gram-negative bacteria from Gram-positive bacteria. Figure 1C,D shows the optical microscopic images of *E. coli* O157:H7 without and with the addition of the optimal concentration of H_2_O_2_, respectively. A ring derived from bacterial cells was observed in the optical image in Figure 1C. However, the bacterial cells were disrupted after the addition of H_2_O_2_ (11.2 mM, 2 µL), and the ring, consisting of bacterial cells, had disappeared (Figure 1D). The results were further confirmed by using transmission electron microscopy (TEM). Figure 1E,F show the TEM images of *E. coli* O157:H7 obtained before and after the addition of H_2_O_2_, respectively. Intact *E. coli* O157:H7 cells were clearly observed prior to the addition of H_2_O_2_ (Figure 1E). However, debris derived from *E. coli* O157:H7 mainly dominated the TEM image (Figure 1F), indicating that the disruption of bacterial cells occurred after the addition of H_2_O_2_. These results suggested that H_2_O_2_ has permeated and disrupted the bacterial cells, resulting in the release of biomolecules, such as peroxidase and catalases, triggering enzymatic reactions.

### 3.2. Optimization of Experimental Parameters

We further investigated the optimal experimental conditions needed for bacterial endogenous peroxidase reactions in the presence of H_2_O_2_, with TMB as the substrate. *E. coli* O157:H7 was used as the model bacterium. Figure S1A shows the photograph of the samples containing *E. coli* O157:H7 (0.2 mL; OD_600_ = ~1), TMB (1.25 mM), H_2_O_2_ at different concentrations (1.4–722 mM), and with sulfuric acid (2 M, 2 µL) to stop the reaction. Figure S1B shows the corresponding UV–Vis absorption spectra of the resultant samples. The yellow color intensified as the concentration of H_2_O_2_ increased, but the sample became paler as the concentration of H_2_O_2_ exceeded 90 mM and decreased to less than 2.4 mM. The optimal concentration of H_2_O_2_ was ~11.2 mM. Presumably, alkyl hydroperoxide reductase (AhP), the major bacterial peroxidase for peroxidase reactions, was triggered at a lower concentration of H_2_O_2_ [37,38]. However, AhP was inactivated at a high concentration of H_2_O_2_ because of a limited cell capacity to provide electrons for H_2_O_2_ reduction [39]. The reaction was conducted and examined under different pH conditions to investigate the pH effects. Figure S1C shows the resultant UV–Vis absorption spectra of the samples containing *E. coli* O157:H7 (OD_600_ = 1, 0.2 mL). The spectra were obtained after reaction with H_2_O_2_ (11.2 mM) and TMB (1.25 mM) at different pH values for 25 min, with the addition of sulfuric acid to stop the reaction. The inset shows the corresponding photographs of the resultant samples. The samples became yellow at pH 3, 4, and 5. No apparent color change was observed at pH 2, 6, and 7. That is, the optimal reaction occurred at pH 3. Presumably, H_2_O_2_ effectively penetrated the bacterial cells and reacted substantially with peroxidases under acidic conditions [40,41,42]. However, when the reaction solution was excessively acidic (pH 2), the enzymatic activity of peroxidase was suppressed. Therefore, no reactions occurred at pH 2. Thus, pH 3 was selected as the optimal reaction condition in subsequent studies. Moreover, the optimal reaction temperature was observed at 25 °C (Figure S1D).

The optimal reaction time for endogenous enzymatic reactions in the samples including *E. coli* O157:H7 and *S. aureus* (SI Figure S2A) was determined. The highest absorbance band was obtained after *E. coli* O157:H7 was reacted with TMB and H_2_O_2_ (11.2 mM) for 25 min (SI Figure S2A). Moreover, many bubbles derived from oxygen were observed from the sample containing *S. aureus* that was reacted with H_2_O_2_ (180 mM) for only 5 min (SI Figure S2B). These results indicated that the optimal reaction time for visual assessment of the color change from the endogenous peroxidase reaction derived from *E. coli* O157:H7 was ~25 min, whereas the optimal reaction time for visual assessment of the presence of *S. aureus* according to bubble formation was only ~5 min.

SI Figure S3 shows the photographs of the samples (0.2 mL) containing *S. aureus* (OD_600_ = ~1; Figure S3A) and *E. coli* O157:H7 (OD_600_ = ~1; Figure S3B) obtained after reaction with H_2_O_2_ at different concentrations (5.6–2890 mM), prepared in phosphate buffer at pH 3. Bubbles from the samples containing *S. aureus* were clearly observed after adding H_2_O_2_ at concentrations of 90–2890 mM, indicating that the activities of bacterial endogenous catalases were induced at a high concentration (≥ 90 mM) of hydrogen peroxide (Figure S3A). The bubbles were not observed in the samples containing *E. coli* O157:H7 after the addition of H_2_O_2_ at concentrations of ≤ 180 mM (Figure S3B). Nevertheless, intense color changes were visible in the samples containing *E. coli* O157:H7 that were reacted with TMB in the presence of H_2_O_2_ at concentrations of ~6–90 mM (cf. Figure S1A). According to the results shown in SI Figures S1 and S3, we concluded that the optimal concentration of H_2_O_2_ for revealing endogenous peroxidase activity derived from *E. coli* O157:H7 was ≤ 90 mM, whereas the concentration of H_2_O_2_ for triggering endogenous catalase activity derived from *S. aureus* was ≥ 90 mM. That is, endogenous peroxidase or catalase reactions could be observed by adjusting the concentration of H_2_O_2_ in the enzymatic reactions. Therefore, either Gram-positive, catalase-positive bacteria or Gram-negative bacteria can be distinguished, based on the color change or bubble formation, respectively, by adjusting the concentration of H_2_O_2_ in the endogenous enzymatic reaction.

Moreover, we also selected two more Gram-positive bacteria, i.e., *B. cereus* and *E. faecalis*, as the model samples, to examine whether bubbles were formed in the addition of H_2_O_2_. SI Figure S4A shows the resultant photograph of the samples obtained after reacting with H_2_O_2_. Apparently, many bubbles were formed in the sample containing *B. cereus*, whereas bubbles were barely observed in the sample containing *E. faecalis*. This was understandable because *E. faecalis* is generally considered as a catalase-negative bacterium and may show weak catalase-positive activity only in specific conditions [43]. The results indicated that our method can be used to realize the presence of catalase-positive bacteria. In addition, two more Gram-negative bacteria, i.e., *P. aeruginosa* and *K. pneumoniae*, were selected as the model samples for endogenous peroxidase reactions. SI Figure S4B shows the resultant photographs of these two bacterial samples obtained after reacting with TMB in the presence of H_2_O_2_, followed by the addition of sulfuric acid to stop the reaction. Apparently, the color of the resultant samples became yellow, indicating the presence of peroxidase existing in these two Gram-negative bacteria. That is, our method can also be used to indicate the presence of these two Gram-negative bacteria, based on the color change. This is understandable because Gram-negative bacteria generally contain abundant peroxidases. These results indicated the suggested method can be used to rapidly distinguish the presence of Gram-negative bacteria. However, if the results showed no color change and no bubble formation, one should not exclude the possibility of the presence of Gram-positive, catalase negative bacteria. Moreover, distinguishing among different Gram-positive bacterial strains or different Gram-negative bacteria by the current method is not possible.

### 3.3. Examination of the Lowest Detectable Concentration by the Naked Eye

**Figure 2:**
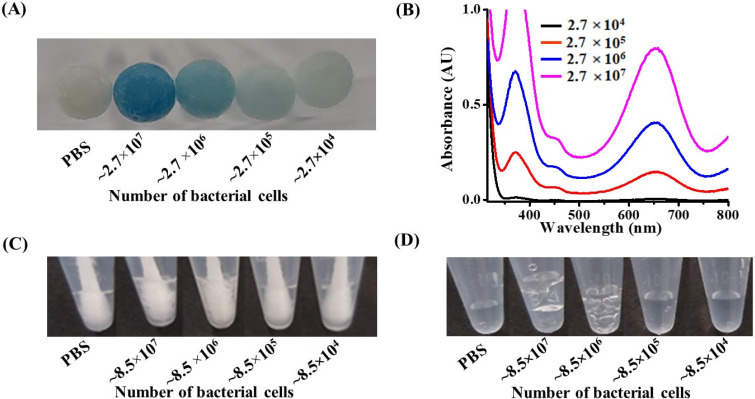
(**A**) Photograph of the cotton swabs obtained after imbuing with the samples (10 µL) containing *E. coli* O157:H7 at concentrations of ~5.4 × 10^5^–~5.4 × 10^8^ cfu mL^−1^ of bacterial cells, prepared at pH 3, followed by dipping into a reagent droplet (50 µL) consisting of TMB (1.25 mM) and H_2_O_2_ (11.2 mM) at pH 3. (**B**) UV–Vis absorption spectra of the bacterial samples (0.2 mL) containing *E. coli* O157:H7 at concentrations of ~5.4 × 10^5^–~5.4 × 10^8^ cfu mL^−1^ obtained after reaction with hydrogen peroxide (~11.2 mM) at pH 3 (0.2 mL) for 25 min. (**C**) Photograph of the cotton swabs obtained after sampling *S. aureus,* at concentrations of ~8.5 × 10^5^–~8.5 × 10^8^ cfu mL^−1^, from the samples (0.1 mL) prepared in the phosphate buffer at pH 3, followed by the reaction with H_2_O_2_ (180 mM, 0.1 mL) at pH 3. (**D**) Photograph of the bacterial samples (0.1 mL) containing *S. aureus* at concentrations of ~8.5 × 10^5^–~8.5 × 10^8^ cfu mL^−1^ cells obtained after reaction with hydrogen peroxide (180 mM, 0.1 mL) prepared in the phosphate buffer at pH 3 for 5 min.

We further investigated the lowest detectable bacterial cell concentration using the developed method. *E. coli* O157:H7 and *S. aureus* were used as the model bacteria. Figure 2A shows the photograph of the cotton swabs imbued with *E. coli* O157:H7 samples (50 µL containing ~2.7 × 10^4^–2.7 × 10^7^ cells), prepared in the phosphate buffer at pH 3 containing TMB (1.25 mM) and H_2_O_2_ (11.2 mM). An apparent blue color appeared on the swab with the highest number of bacterial cells. When the amount of *E. coli* O157:H7 was dropped to ~2.7 × 10^5^, a pale bluish color on the swab was still visible, indicating that the lowest visualizable amount was ~10^5^ bacterial cells. Figure 2B shows the UV–Vis absorption spectra of *E. coli* J96 sample in the phosphate buffer, with the same treatment as shown in Figure 2A. The maximum absorption band appeared at a wavelength of ~650 nm, in which the intensity was proportional to the cell concentration of *E. coli* O157:H7. Cotton swabs were used when sampling *S. aureus* from the samples (50 µL) containing 8.5 × 10^4^–8.5 × 10^7^ cells, prepared in the phosphate buffer at pH 3 and then immersed in a reagent (0.1 mL) containing H_2_O_2_ (180 mM) prepared in the phosphate buffer at pH 3. Figure 2C shows the resultant photograph, in which the bubbles containing oxygen increased with the concentration of *S. aureus* because of the endogenous catalase reaction (SI Scheme S1). In contrast, the endogenous reaction derived from *S. aureus* was directly conducted in a liquid reagent containing TMB/H_2_O_2_. Figure 2D shows the photographs of the *S. aureus* samples (0.1 mL), containing ~8.5 × 10^4^–8.5 × 10^7^ cells, after reaction with H_2_O_2_ (180 mM) for 5 min. Bubbles were observed in the samples containing *S. aureus* with a cell number of ≥ ~10^6^ cells, similar to those observed in Figure 2C. The results indicated that it was possible to visually assess the presence of Gram-positive bacteria such as *S. aureus* in the sample, based on the observation of bubbles.

### 3.4. Examination of Selectivity

**Figure 3:**
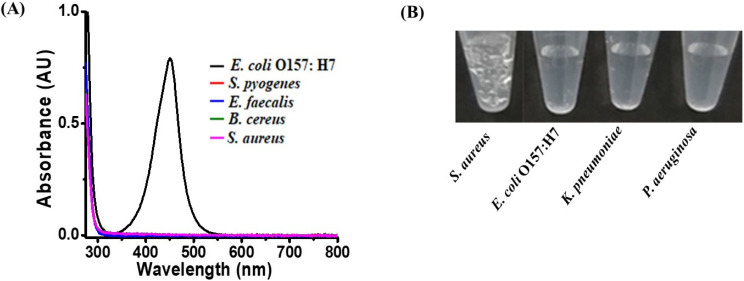
Examination of selectivity. (**A**) UV–Vis absorption spectra of the samples (0.2 mL) containing *E. coli* O157:H7, *S. aureus, B. cereus, S. pyogenes*, and *E. faecalis* prepared in the phosphate buffer at pH 3; spectra were obtained after reaction with TMB (1.25 mM) in the presence of H_2_O_2_ (~11.2 mM) for 25 min, followed by the addition of sulfuric acid (2 M, 2 µL) to stop the reaction. All the model bacteria had the cell concentration of OD_600_ equal to ~1. (**B**) Photograph of the samples (0.2 mL) containing *S. aureus*, *E. coli* O157:H7, *K. pneumoniae*, and *P. aeruginosa* (from left to right) prepared in the phosphate buffer at pH 3 and obtained after reaction with H_2_O_2_ (180 mM) for 5 min. All the model bacteria had a cell concentration of OD_600_ equal to ~1.

The performance of the current approach in discriminating between Gram-positive and Gram-negative bacteria was evaluated. Gram-positive bacteria, including *S. aureus*, *S. pyogenes*, *B. cereus*, and *E. faecalis*, and Gram-negative bacteria, including *E. coli* O157:H7, *P. aeruginosa*, and *K. pneumoniae*, were used as the model bacteria. Figure 3A shows the UV–Vis absorption spectra of the samples (0.2 mL, OD_600_ = ~1) containing *E. coli* O157:H7, *S. aureus*, *B. cereus*, *S. pyogenes*, and *E. faecalis* with the same OD_600_ at ~1, after reaction with TMB (1.25 mM), in the presence of H_2_O_2_ (11.2 mM) prepared in phosphate buffer at pH 3 and the subsequent addition of sulfuric acid to stop the reaction. Only the sample containing *E. coli* O157:H7 showed an absorption band at a wavelength of ~450 nm. The samples containing Gram-positive bacteria did not have any apparent absorption band, indicating that they did not have sufficient peroxidase to carry out enzymatic reactions. Figure 3B shows the photographs of the samples (0.2 mL) containing *S. aureus*, *E. coli* O157:H7, *K. pneumoniae*, and *P. aeruginosa* that were obtained after reaction with H_2_O_2_ (180 mM) prepared in phosphate buffer at pH 3. Apparently, only the sample containing *S. aureus* showed observable bubbles, indicating that the bacterial endogenous catalase reaction was triggered. The rest of the samples did not show any bubbles, indicating that there was not good catalase activity with H_2_O_2_ (180 mM). That is, the results demonstrated that Gram-positive or Gram-negative bacteria in a sample could be detected by adding different concentrations of H_2_O_2_. Bubble formation and color changes can be used as indicators for detecting Gram-positive bacteria and Gram-negative bacteria, respectively.

### 3.5. Analysis of Different Strains of E. coli

**Figure 4:**
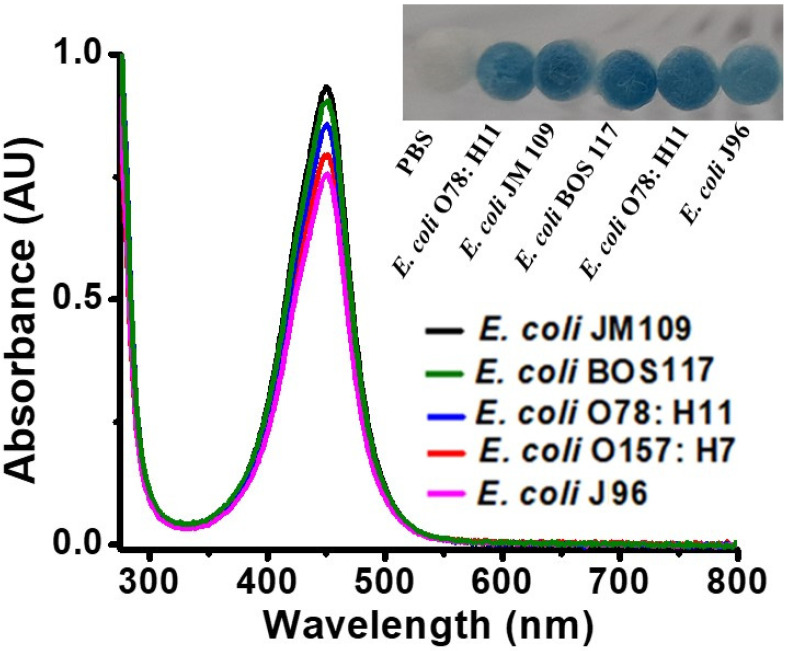
UV–Vis absorption spectra of the samples (0.2 mL) containing different *E. coli* strains (OD_600_ of ~1), obtained after reaction with H_2_O_2_ (11.2 mM) in the presence of TMB (1.25 mM) at pH 3, followed by the addition of sulfuric acid (2 M, 2 µL) to stop the reaction. (Inset) shows the photograph of the cotton swabs containing *E. coli* strains (OD_600_ of ~1) obtained after reaction with H_2_O_2_ (11.2 mM) in the presence of TMB (1.25 mM) at pH 3. The reaction was conducted at room temperature (~25 °C).

Different strains of *E. coli,* including *E. coli* JM109, *E. coli* J96, *E. coli* O78:H11, and *E. coli* BOS 117 were also used as the model bacteria, to examine whether the current approach was effective for these different *E. coli* strains. *E. coli* O157:H7 was also examined again for comparison. The optimal reaction conditions obtained above were applied to conduct the reaction. The inset in Figure 4 shows the photographs of the resultant samples of different *E. coli* strains were obtained after reacting with TMB in the presence of hydrogen peroxide. All the cotton swabs became blue, indicating that the method can be used to sense the presence of different *E. coli* strains. Figure 4 shows the resultant UV–Vis absorption spectra of these *E. coli* samples, obtained after the reaction with TMB in the presence of hydrogen peroxide, followed by the addition of sulfuric acid to stop the reaction. The maximum absorbance band at the wavelength of 450 nm among different *E. coli* strains looked similar, indicating that these *E. coli* strains had similar responses to the endogenous peroxidase reaction.

### 3.6. Examination of Interference Effects

**Figure 5:**
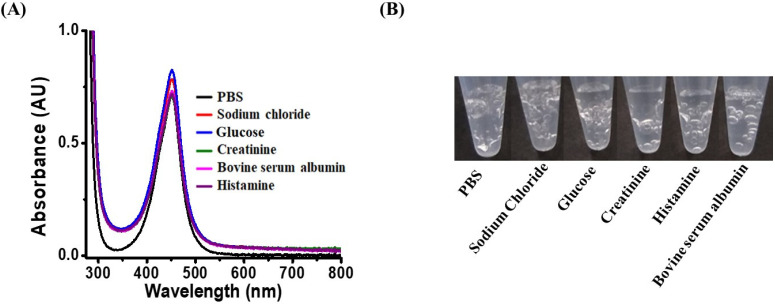
Examination of interference species effects. (**A**) UV–Vis absorption spectra of the samples (0.2 mL) containing *E. coli* O157:H7 (OD_600_ = ~1) with the inferences species including sodium chloride (1 mM), potassium chloride (1 mM), creatinine (1 mM), bovine serum albumin (1 mM), and histamine (1 mM) obtained after reaction with H_2_O_2_ (11.2 mM) and TMB (1.25 mM) for 25 min followed by the addition of sulfuric acid (2 M, 2 μL) to stop the reaction. All the samples were prepared in the phosphate buffer at pH 3. (**B**) Photograph of the samples (0.2 mL) containing *S. aureus* (OD_600_ = 1) with interferences including sodium chloride (1 mM), potassium chloride (1 mM), creatinine (1 mM), bovine serum albumin (1 mM), and histidine (1 mM), obtained after the reaction with H_2_O_2_ (180 mM) for 5 min.

Whether the results were affected by the presence of sodium chloride, potassium chloride, creatinine, bovine serum albumin, and histamine, which are commonly present in real-world samples, was determined by conducting sensing experiments in the presence of these species. Because the concentrations of these selected interference species usually are not over 1 mM in real samples, 1 mM of each interference species was used for the preparation of the samples. *E. coli* O157:H7 was used as the model bacterium. Figure 5A shows the UV–Vis absorption spectra of the samples containing *E. coli* O157:H7 after the addition of sodium chloride (1 mM), potassium chloride (1 mM), creatinine (1 mM), bovine serum albumin (1 mM), and histidine (1 mM). The intensities of the maximum absorption at the wavelength of ~450 nm in all the absorption spectra were highly similar, indicating that the reaction was not considerably affected by the presence of the additives. Whether the bacterial catalase reaction itself was affected was determined by examining samples containing *S. aureus* and the same interference species as used above. Figure 5B shows the photographs of the samples. Apparently, all the samples generated observable bubbles, indicating that the additives did not affect the endogenous catalase reactions.

Although this study emphasized the discrimination of Gram-positive bacteria and Gram-negative bacteria, we also considered the interference from other microorganisms such as fungi. For example, *A. niger*, which generates black spores, also contains abundant catalases [44]. Thus, bubble formation may be observed when a number of spores are present in the sample using our method. Figure S6A shows the photograph of the samples including the mixtures of *E. coli* O157:H7 (OD_600_ of ~1) and *A. niger* spores, at concentrations of OD_600_ of ~1, ~0.1, and ~0.01 (left to right), as obtained after reaction with TMB in the presence of H_2_O_2_ (~11.2 mM). Figure S6B shows the same samples used for Figure S6A with the further addition of H_2_O_2_ (180 mM). The results shown in Figure S6A,B bore a close resemblance. Apparently, the samples became blue owing to the presence of *E. coli* O157:H7, whereas bubbles were observed in some sample vials. The number of bubbles decreased as the concentration of *A. niger* decreased. Moreover, black spores were apparently visible in the samples containing *A. niger* at concentrations of OD_600_ of ~1 and ~0.1. Although bubbles were formed in the samples containing *A. niger*, the concentration of *A. niger* needed to reach OD_600_ ≥ ~0.1 to be observed easily. On the other hand, we were able to realize the presence of black spores at a concentration of OD_600_ ≥ ~0.1 by the naked eye. However, unlike fungi, the assay developed in this study was required to determine the presence of Gram-positive and Gram-negative bacteria in the samples, owing to the small size and invisibility of bacterial cells. The results indicated that the presence of *A. niger* at a concentration higher than ~0.1 in the sample can also generate bubbles using our method, which may lead to misdiagnosis of the presence of Gram-positive bacteria. Nevertheless, one can realize the presence of fungi at OD_600_ higher than ~0.1 without conducting additional endogenous enzymatic reactions, because of the visible fungal spores.

### 3.7. Analysis of Real Samples

**Figure 6:**
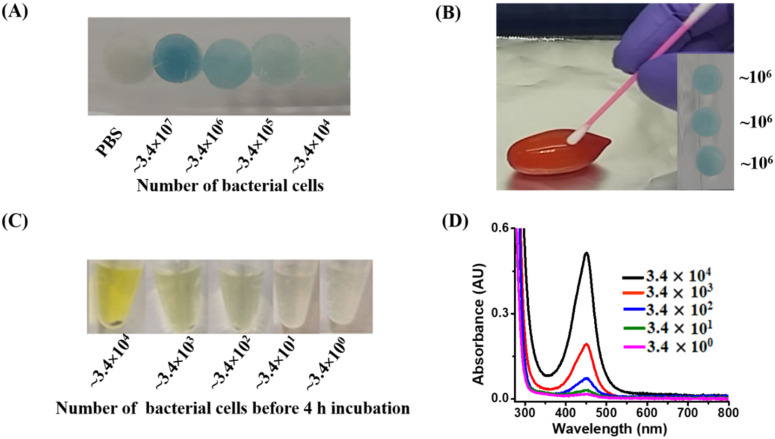
Detection of bacteria from bacterium-contaminated cherry tomatoes. (**A**) Photograph of the cotton swabs obtained after sampling bacteria from the cherry tomatoes smeared with PBS only, and ~3.4 × 10^7^–3.4 × 10^4^ *E. coli* O157:H7 cells (left to right), followed by dipping into a reagent (50 µL) containing H_2_O_2_ (~11.2 mM) and TMB (1.25 mM) at pH 3. (**B**) Photograph of the as-prepared tomato that was sampled by a cotton swab. The photograph inset on the right-hand side shows the three swabs obtained after sampling bacteria from the surface of three individual tomatoes smeared with ~10^6^ cells of *E. coli* O157:H7, followed by immersion in a reagent droplet (50 µL) containing H_2_O_2_ and TMB at pH 3. (**C**) Photograph of the samples obtained after collecting bacteria from the cherry tomatoes smeared with ~3.4 × 10^4^–~3 *E. coli* O157:H7 cells (left to right), followed by incubation in a growth broth (0.4 mL) for 4 h and then reacted with the reagent (0.2 mL) containing H_2_O_2_ (~11.2 mM) and TMB (1.25 mM) at pH 3, and the addition of sulfuric acid (2 M, 2 µL). (**D**) The corresponding UV–Vis absorption spectra of the samples obtained in (**C**).

We further investigated the feasibility of using our approach in the direct detection of bacteria in simulated real samples. *E. coli* O157:H7 may contaminate vegetables or fruits, such as tomatoes. Thus, cherry tomatoes were selected as model samples for the preparation of simulated real samples. We smeared trace bacteria (*E. coli* O157:H7) on the surface of an intact tomato. The samples containing *E. coli* O157:H7 with different concentrations were prepared by serially diluting an *E. coli* O157:H7 suspension, with an OD_600_ value of 1, four times with a dilution factor of 10. Figure 6A shows the photographs of the cotton swabs, obtained after *E. coli* O157:H7 samples (10 µL) with a cell number ranging from ~3.4 × 10^4^ to ~3.4 × 10^7^ cells were collected and immersed in a reagent droplet (50 µL) containing H_2_O_2_ and TMB. The experimental details are described in the Experimental Section. The blue color of the swab became pale as the number of bacterial cells decreased. We were still able to see a pale bluish discoloration on the swab at a cell number of ~10^5^. We further prepared three cherry tomatoes smeared with trace bacteria (*E. coli* O157:H7; 50 µL; ~6.8 × 10^7^ cfu mL^−1^; Figure 6B). After the bacterial sample was dried on the cherry tomato, a cotton swab was used in collecting bacteria from the surface. The cotton swab was then immersed in a reagent droplet (50 µL) containing H_2_O_2_ (11.2 mM) and TMB (1.25 mM) at pH 3. The inset photographs show the resultant swabs from three replicated experiments. Compared with the results shown in Figure 6A, the bacterial sample from the peel of an individual cherry tomato was ~10^6^ cells. That is, the current approach can be used in roughly estimating bacterial cell numbers according to color changes from the results obtained in standard samples. However, directly observing the presence of *E*. *coli* O157: H7 with a cell number below ~10^5^ cells is impossible using this approach. Nevertheless, given that *E. coli* can divide into two cells every 4–20 min under nutrient-rich and aerobic environmental conditions [45], 4 h of incubation should be enough to generate sufficient bacterial cells for visual assessment with our method. Thus, the sample was further incubated in a growth medium for another 4 h before the enzymatic reaction. Figure 6C shows the photographs of the samples obtained from the surfaces of intact cherry tomatoes smeared with *E. coli* O157:H7, with cell numbers of ~3.4 × 10^4^, ~3.4 × 10^3^, ~3.4 × 10^2^, and ~3, followed by 4-hour incubation and peroxidase reactions. Sulfuric acid was used to stop the reaction. Figure 6D shows the corresponding UV–Vis absorption of the same samples in Figure 6C. According to these results, the lowest detectable cell number was ~34. In addition, we also investigated whether the sensitivity of our method toward *S. aureus* could be further improved by incubating the swabs that were tainted with *S. aureus* at different cell numbers, followed by 4-hour incubation and endogenous enzymatic reactions. SI Figure S5 shows a photograph of the resultant samples. Apparently, air bubbles were still observable in the samples with initial bacterial cells to ≥ ~8.5 × 10^3^. No bubbles were observed when the cell number was reduced to ~850. The analysis time, including bacterial incubation, was ~4.5 h, which was considerably shorter than that needed for conventional overnight culture bioassays. However, the detectable bacterial cell numbers in the samples containing *E. coli* O157:H7 and *S. aureus* were reduced to few tens and few thousands, respectively.

**Figure 7:**
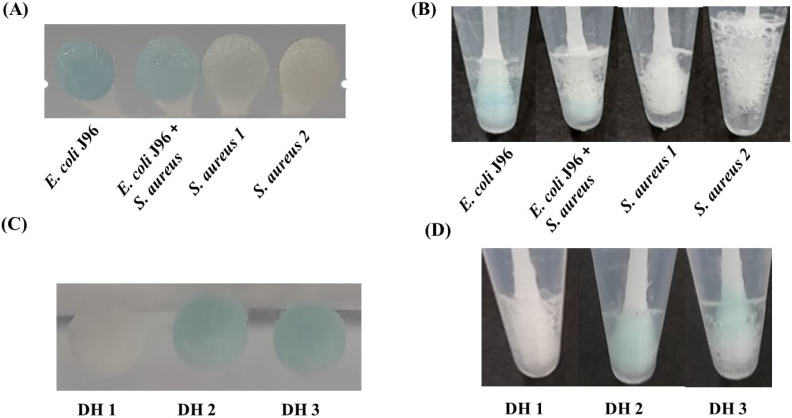
Detection of bacteria from door handles. (**A**) Photograph of the cotton swabs obtained after sampling bacteria from door handles smeared with the bacterial samples, including *E. coli* J96 (2.7 × 10^5^ cells), the mixture of *E. coli* J96 (~2.7 × 10^5^ cells) and *S. aureus* (~8.5 × 10^7^ cells), and two *S. aureus* samples with different cell numbers (~8.5 × 10^5^ cells (*S. aureus* 1) and ~8.5 × 10^7^ cells (*S. aureus* 2)), followed by dipping into a reagent (50 µL) containing H_2_O_2_ (11.2 mM) and TMB (1.25 mM) at pH 3. (**B**) Photograph of the cotton swabs from Panel A, obtained after further reaction with a reagent containing H_2_O_2_ (180 mM, 0.1 mL) at pH 3. (**C**) Photograph of the swabs used to collect bacteria from the door handles from the restroom, obtained after reaction with the reagent (50 µL) containing TMB (1.25 mM) and H_2_O_2_ (~11.2 mM). (**D**) Photograph of the cotton swabs from Panel C, obtained after further reaction with H_2_O_2_ (180 mM, 0.1 mL) at pH 3.

*E. coli* and *S. aureus* are common pathogens and may be commonly found on door handles. Thus, door handles contaminated with *E. coli* J96 and *S. aureus* were prepared. We smeared trace amounts of bacteria, including *E. coli* J96 and *S. aureus*, on the surfaces of door handles. The experimental details are described in Section 2.7. Figure 7A shows the photographs of the cotton swabs obtained from the door handle samples spiked with *E. coli* J96, a mixture of *E. coli* J96 and *S. aureus*, and two samples of *S. aureus*. All samples were reacted with a reagent (50 µL) containing H_2_O_2_ (11.2 mM) and TMB (1.25 mM) prepared in phosphate buffer at pH 3 for 25 min. Figure 7B shows the photograph of the resultant cotton swabs that were further reacted with a high concentration (180 mM) of H_2_O_2_ solution (0.1 mL) at pH 3. The results from the first step revealed the presence of *E. coli* J96 because the swabs showed a bluish color, whereas the results from the second step indicated the presence of *S. aureus,* owing to bubble formation. Our method can thus be used for detecting pathogenic bacteria. Furthermore, determining the presence of either Gram-positive bacteria or Gram-negative bacteria is possible by using the two-step method for the results. We took samples directly from the door handles (DH1, DH2, and DH3) of the restroom in our building and determined whether bacteria were present on the samples with our two-step method. Figure 7C shows the photograph of the resulting three cotton swabs obtained after our method was used on the samples. Apparently, the swabs from DH2 and DH3 became bluish, indicating the presence of Gram-negative bacteria with a cell number of > 10^5^ (cf. Figure 2A). Figure 7D shows the results obtained after treating the same swabs shown in Figure 7C with a high concentration of H_2_O_2_ (180 mM, 0.1 mL). Apparently, only the swabs from DH1 and DH3 generated visible bubbles, indicating that DH1 and DH3 contained Gram-positive bacteria with a cell number of > ~10^6^ cells (cf. Figure 2D). That is, DH1 contained Gram-positive bacteria with a cell number of > ~10^5^, whereas DH2 only contained Gram-negative bacteria with a bacterial cell number of > ~10^6.^ Moreover, DH3 contained Gram-positive and Gram-negative bacteria. To further confirm the presence of bacteria on these door-handle samples, the other cotton swabs were used to collect further samples from these three door handles. SI Figure S7A–C shows the photographs of the agar plate of the cultured samples collected from these three door-handles after incubation for 14 h. Apparently, many bacteria grew on the sample collected from DH1 (SI Figure S7A). More than one type of bacterial colony appeared in the samples collected from DH2 (SI Figure S7B) and DH3 (SI Figure S7C). These results demonstrated that using our two-step method for rapidly detecting pathogenic bacteria with the naked eye is possible.

## 4. Conclusions

Conventional Gram-staining methods for distinguishing Gram-positive from Gram-negative bacteria require freshly harvested bacterial samples. Thus, overnight culture is generally needed for the preparation of bacterial samples. Therefore, rapid identification of Gram-negative or Gram-positive bacteria was limited because of this requirement. A rapid method that can be used in distinguishing Gram-negative bacteria from Gram-positive bacteria by exploiting the bacterial endogenous peroxidase or catalase reactions has been successfully demonstrated in this study. The developed method possesses several advantages, including being label-free and offering good sensitivity and high selectivity. Moreover, the developed method has a considerably shorter analysis time than existing methods used for distinguishing Gram-negative bacteria from Gram-positive bacteria, given that overnight culture is not required. Only a cotton swab and a few chemical reagents are sufficient to complete the sensing method. Moreover, the results can be visually assessed without the use of any instrumentation, if the bacterial cell number is higher than ~10^5^–10^6^. However, the developed method can only be used to discriminate between Gram-positive and Gram-negative bacteria. The capacity for identification among different bacteria is insufficient. The method can be further improved by using affinity-based approaches that have the capability to respond to specific bacteria. Nevertheless, owing to its simplicity, the developed method should have the potential for use in real-world applications. On the basis of similar operation principles, the developed method can be potentially extended to applications in rapid diagnostics for discriminating Gram-positive and Gram-negative bacteria from bacterium-infected skin or wounds. Given that the antibiotics used to treat infections caused by Gram-positive and Gram-negative bacteria are generally different, minimizing the misuse and overuse of antibiotics is thus possible. Therefore, the speed of emerging of antibiotic-resistant bacterial strains can be reduced.

## Supplementary Materials

The following are available online at https://www.mdpi.com/article/10.3390/bios11090317/s1, Scheme S1: Scheme S1. Enzymatic reactions. Figure S1: Examination of the optimal experimental conditions. Figure S2: Examination of the optimal reaction time. Figure S3: Optimization of the concentration of hydrogen peroxide in the catalase reactions. Figure S4: Examination of endogenous enzymatic reactions of four other model bacteria, Figure S5: Examination of the limit of detection of S. aureus-based endogenous enzymatic reactions, Figure S6: Examination of endogenous enzymatic reactions of the mixture of *E. coli* and *A. niger*. Figure S7: Photographs of the agar plates inoculated with the samples collected from door handles.
