# Supplementary material for: Rapid Detection of Pathogenic Bacteria by the Naked Eye
Source: Biosensors (Basel). 2021 Sep 6;11(9):317. doi: 10.3390/bios11090317 (PMC8469438; doi:10.3390/bios11090317)
Supplement: Supplementary file 1 [file biosensors-11-00317-s001.zip › biosensors-1347407-supplementary.pdf]

biosensors

Article

# Rapid Detection of Pathogenic Bacteria by the Naked Eye

**Karthikeyan Kandasamy [1,2], Miftakhul Jannatin [1,2] and Yu-Chie Chen [1,2,*]**

[1] Department of Applied Chemistry, National Chiao Tung University, Hsinchu 300, Taiwan; mirokarthik.c@nycu.edu.tw (K.K.); miftakhulj.ac07g@nctu.edu.tw (M.J.)
[2] Department of Applied Chemistry, National Yang Ming Chiao Tung University, Hsinchu 300, Taiwan
* Correspondence: yuchie@nctu.edu.tw; Tel.: +886-3-5131527; Fax: +886-3-5723764

## Additional Scheme.

**Scheme 1.** Enzymatic reactions. Peroxidase reaction by using 3, 3′, 5, 5′-tetramethyl benzidine as the substrate in the presence of hydrogen peroxide and catalase reaction in the presence of hydrogen peroxide.

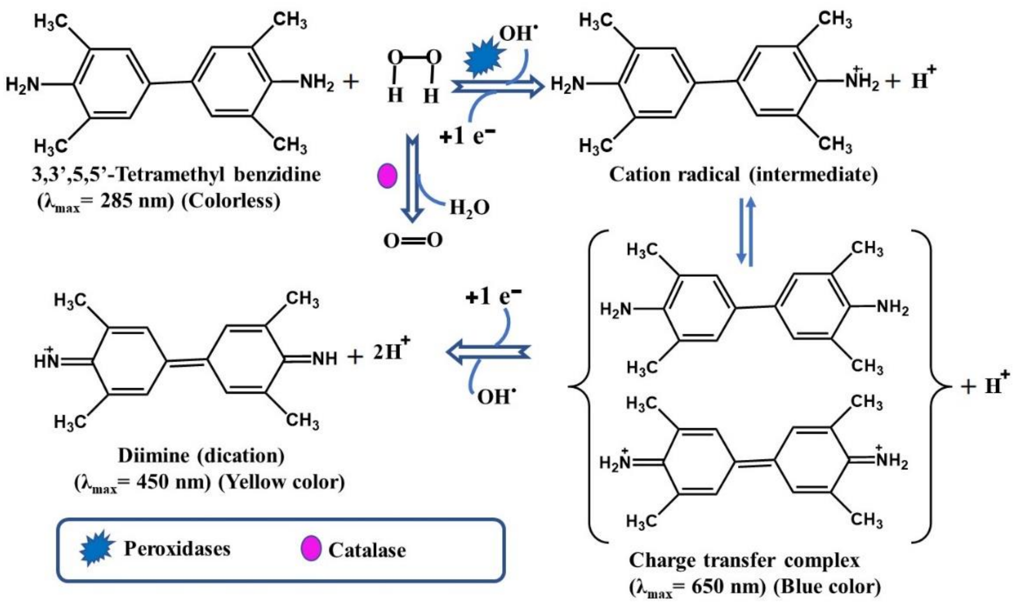


## Additional Figures.

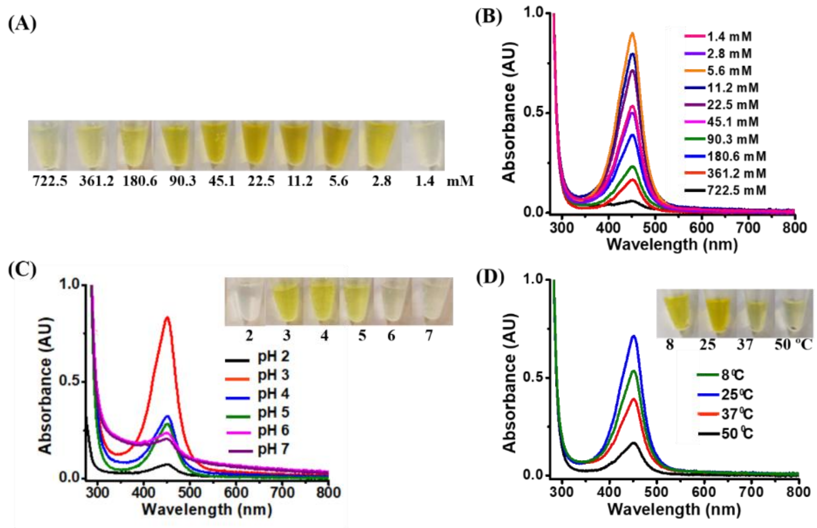


**Citation:** Kandasamy, K.; Jannatin, M.; Chen, Y.-C. Rapid Detection of Pathogenic Bacteria by the Naked Eye. *Biosensors* **2021**, *11*, 317. https://doi.org/10.3390/bios11090317

Received: 3 August 2021
Accepted: 2 September 2021
Published: 6 September 2021

**Publisher's Note:** MDPI stays neutral with regard to jurisdictional claims in published maps and institutional affiliations.

**Copyright:** © 2021 by the authors. Submitted for possible open access publication under the terms and conditions of the Creative Commons Attribution (CC BY) license (http://creativecommons.org/licenses/by/4.0/).

**Figure S1.** Examination of the optimal experimental conditions. (A) Photographs of the samples containing *E. coli* O157:H7 ($OD_{600}$= 1, 0.2 mL) obtained after reaction with $H_2O_2$ (1.41-722 mM) and TMB (1.25 mM) in the phosphate buffer at pH 3 for 25 min at room temperature (~25 $^{o}$C) followed by the addition of sulfuric acid (2 M, 2 μL). (B) The corresponding UV-Vis absorption spectra to the samples shown in Panel A. (C) UV-Vis absorption spectra of the samples containing *E. coli* O157:H7 ($OD_{600}$= 1, 0.2 mL) obtained after reaction with $H_2O_2$ (11.2 mM) and TMB (1.25 mM) at different pH values (pH 2-8) for 25 min followed by the addition of sulfuric acid (2 M, 2 μL) at room temperature. (Inset) Photographs of the corresponding samples shown in Panel C. (D) UV-Vis absorption spectra of the samples containing *E. coli* O157:H7 ($OD_{600}$= ~1, 0.2 mL) obtained after reaction with $H_2O_2$ (11.2 mM) in the presence of TMB (1.25 mM) prepared in the phosphate buffer at pH 3 for 25 min at different temperatures (8, 25, 37 and 50 $^{o}$C) followed by the addition of sulfuric acid (2 M, 2 μL) at room temperature. The inset shows the corresponding photographs of the resultant samples.

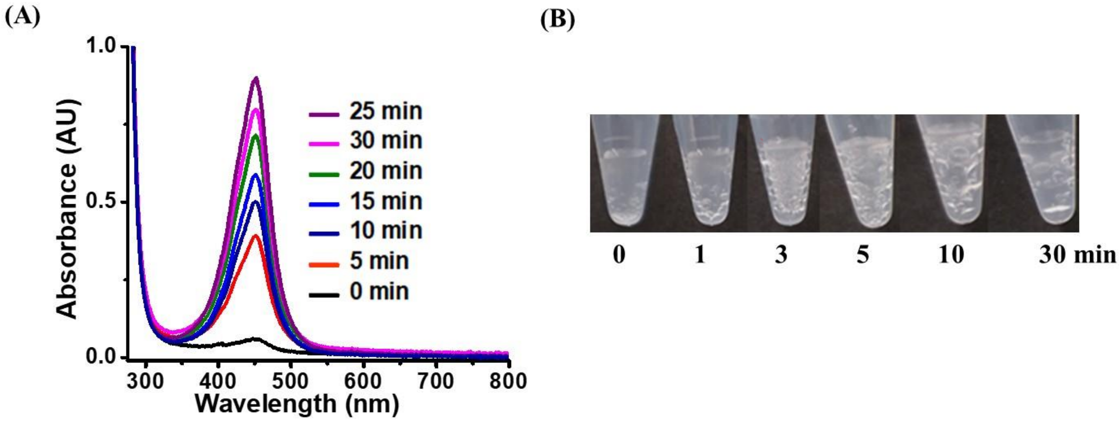


**Figure S2.** Examination of the optimal reaction time. (A) UV-Vis absorption spectra of the samples containing *E. coli* O157:H7 ($OD_{600}$= ~1, 0.2 mL) obtained after reaction with $H_2O_2$ (11.2 mM) and TMB (1.25 mM) prepared in the phosphate buffer (20 mM, pH 3) for different times (0, 5, 10, 15, 20, 25, and 30 min) followed by the addition of sulfuric acid (2M, 2 μL) at room temperature ($OD_{600}$ of 1= ~$6.8\times10^{8}$ cfu $mL^{-1}$). (B) Photograph of the samples containing *S. aureus* ($OD_{600}$ = 1, 0.2 mL) obtained after reaction with $H_2O_2$ (180 mM) prepared in the phosphate buffer at pH 3 for different times (0, 1, 3, 5, 10 and 30 min) ($OD_{600}$ of 1= ~$1.7\times10^{9}$ cfu $mL^{-1}$).

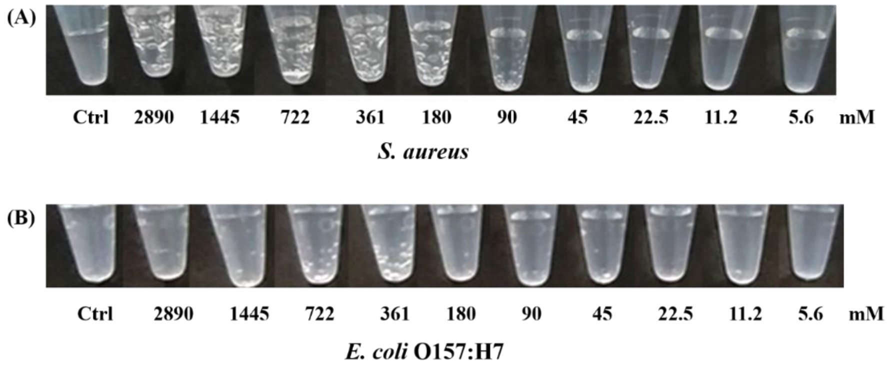


**Figure S3.** Optimization of the concentration of hydrogen peroxide in the catalase reactions. Photographs of the samples containing (A) *S. aureus* ($OD_{600}$= ~1, 0.2 mL) and (B) *E. coli* O157:H7 ($OD_{600}$= ~1, 0.2 mL) obtained after reaction with hydrogen peroxide at different concentrations (5.6-2890 mM) prepared in the phosphate buffer at pH 3 at room temperature (~25 $^{o}$C). The sample vials labeled with Ctrl in Panels (A) and (B) were the samples without the addition of hydrogen peroxide.

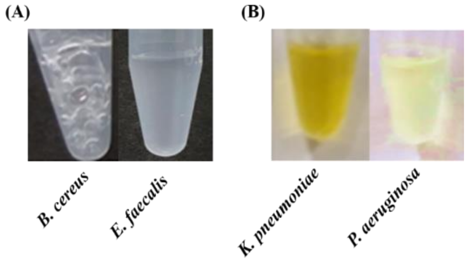


**Figure S4.** Examination of endogenous enzymatic reactions of four other model bacteria. (A) Photograph of the bacterial samples (0.2 mL) including *B. cereus* (left) and *E. faecalis* (right) with the concentration of ($OD_{600}$= ~ 1) obtained after reaction with $H_2O_2$ (180 mM) prepared in the phosphate buffer at pH 3. The reaction was conducted at room temperature. (B) Photograph of the bacterial samples (0.2 mL) including *P. aeruginosa* (left) and *K. pneumoniae* (right) with the concentration of ($OD_{600}$= ~ 1) obtained after reaction with TMB in the presence of $H_2O_2$ at the concentration of 722 mM and 11.2 mM, respectively, prepared in the phosphate buffer at pH 3 followed by the addition of sulfuric acid (2 M, 2 μL) at room temperature.

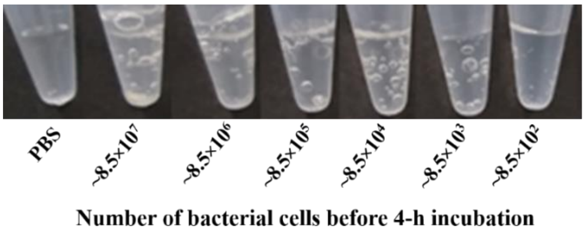


**Figure S5.** Examination of the limit of detection of *S. aureus*-based endogenous enzymatic reactions. Photograph of the samples containing *S. aureus* with cell number of ~ $8.5 \times 10^7$, ~ $8.5 \times 10^6$, ~ $8.5 \times 10^5$, ~ $8.5 \times 10^4$, ~ $8.5 \times 10^3$, and ~ $8.5 \times 10^2$ subjected to 4 h incubation followed by reaction with $H_2O_2$ (180 mM, 0.2 mL) in the phosphate buffer at pH 3. The reaction was conducted at room temperature.

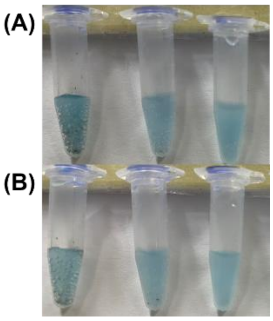


**Figure S6.** Examination of endogenous enzymatic reactions of the mixture of *E. coli* and *A. niger*. Photographs of the samples (0.2 mL) including the mixtures of *E. coli* O157:H7 ($OD_{600}$ of ~1) and *A. niger* spores with the concentrations of $OD_{600}$ of ~1, ~0.1, and ~0.01 (left to right) obtained (A) after reaction with TMB (1.25 mM) in the presence of $H_2O_2$ (~11.2 mM) prepared in the phosphate buffer at pH 3 for 25 min and (B) subsequent addition of $H_2O_2$ (5 μL, 7.6 M) for reaction with another 5 min.

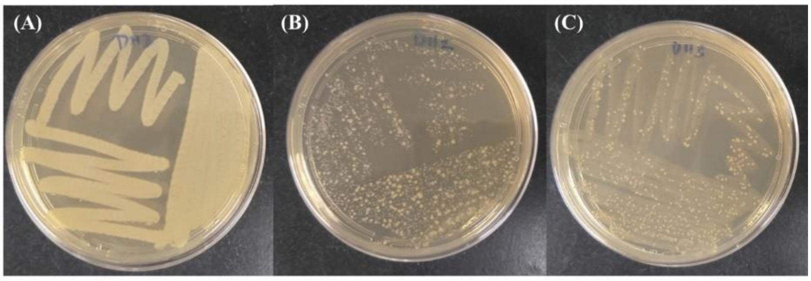


**DH1** **DH2** **DH3**

**Figure S7.** Photographs of the agar plates inoculated with the samples collected from door handles (A) DH1, (B) DH2, and (C) DH3 obtained after overnight culture.
